# Typhoid Fever

**Published:** 1919-12

**Authors:** 


**Typhoid Fever.** By FREDERICK P. GAY, Professor of Pathology in the University of California. Pp. xi., 286. New York: The Macmillan Company. 1918. Price 12s. 6d. net.

This exhaustive and closely-printed work is not intended as a clinical or pathological manual, but as a critical précis of our present knowledge of the development and spread of typhoid and paratyphoid, and of the methods employed in its diagnosis, prevention and treatment. There is a bibliography of over forty pages, which will give some idea of the immense material which it embraces in its far-reaching *conspectus*. The author's scheme is an ambitious one, and he has carried it out with much judgment and success. The book, however, suffers somewhat in that it could not include the completed evidence on the bacteriological and serological aspects of the subject amassed during the Great War. Though there is no index, the schematic table of contents is a good guide to the work, and it may be recommended as a useful book of reference to those desiring to study the complicated problems presented by the epidemiology and pathology of the enteric diseases.

